# Transmission Dynamics of the COVID-19 Epidemic at the District Level in India: Prospective Observational Study

**DOI:** 10.2196/22678

**Published:** 2020-10-15

**Authors:** Suman Saurabh, Mahendra Kumar Verma, Vaishali Gautam, Nitesh Kumar, Akhil Dhanesh Goel, Manoj Kumar Gupta, Pankaj Bhardwaj, Sanjeev Misra

**Affiliations:** 1 Department of Community Medicine and Family Medicine All India Institute of Medical Sciences Jodhpur India; 2 All India Institute of Medical Sciences Jodhpur India

**Keywords:** Epidemiology, SARS-CoV-2, COVID-19, serial interval, basic reproduction number, projection, outbreak response, India, mathematical modeling, infectious disease

## Abstract

**Background:**

On March 9, 2020, the first COVID-19 case was reported in Jodhpur, Rajasthan, in the northwestern part of India. Understanding the epidemiology of COVID-19 at a local level is becoming increasingly important to guide measures to control the pandemic.

**Objective:**

The aim of this study was to estimate the serial interval and basic reproduction number (R_0_) to understand the transmission dynamics of the COVID-19 outbreak at a district level. We used standard mathematical modeling approaches to assess the utility of these factors in determining the effectiveness of COVID-19 responses and projecting the size of the epidemic.

**Methods:**

Contact tracing of individuals infected with SARS-CoV-2 was performed to obtain the serial intervals. The median and 95th percentile values of the SARS-CoV-2 serial interval were obtained from the best fits with the weibull, log-normal, log-logistic, gamma, and generalized gamma distributions. Aggregate and instantaneous R_0_ values were derived with different methods using the EarlyR and EpiEstim packages in R software.

**Results:**

The median and 95th percentile values of the serial interval were 5.23 days (95% CI 4.72-5.79) and 13.20 days (95% CI 10.90-18.18), respectively. R_0_ during the first 30 days of the outbreak was 1.62 (95% CI 1.07-2.17), which subsequently decreased to 1.15 (95% CI 1.09-1.21). The peak instantaneous R_0_ values obtained using a Poisson process developed by Jombert et al were 6.53 (95% CI 2.12-13.38) and 3.43 (95% CI 1.71-5.74) for sliding time windows of 7 and 14 days, respectively. The peak R_0_ values obtained using the method by Wallinga and Teunis were 2.96 (95% CI 2.52-3.36) and 2.92 (95% CI 2.65-3.22) for sliding time windows of 7 and 14 days, respectively. R_0_ values of 1.21 (95% CI 1.09-1.34) and 1.12 (95% CI 1.03-1.21) for the 7- and 14-day sliding time windows, respectively, were obtained on July 6, 2020, using method by Jombert et al. Using the method by Wallinga and Teunis, values of 0.32 (95% CI 0.27-0.36) and 0.61 (95% CI 0.58-0.63) were obtained for the 7- and 14-day sliding time windows, respectively. The projection of cases over the next month was 2131 (95% CI 1799-2462). Reductions of transmission by 25% and 50% corresponding to reasonable and aggressive control measures could lead to 58.7% and 84.0% reductions in epidemic size, respectively.

**Conclusions:**

The projected transmission reductions indicate that strengthening control measures could lead to proportionate reductions of the size of the COVID-19 epidemic. Time-dependent instantaneous R_0_ estimation based on the process by Jombart et al was found to be better suited for guiding COVID-19 response at the district level than overall R_0_ or instantaneous R_0_ estimation by the Wallinga and Teunis method. A data-driven approach at the local level is proposed to be useful in guiding public health strategy and surge capacity planning.

## Introduction

COVID-19 has emerged as the largest pandemic of the 21st century, with 30.7 million confirmed cases and approximately 950,000 deaths worldwide as of September 2020 [1]. India has become the second most affected country worldwide after the United States, with approximately 5.4 million confirmed COVID-19 cases [1]. COVID-19 is an emerging infectious disease, the first case being reported from Wuhan, China, in early December 2019 [2]. Various epidemiological studies are being performed to understand the transmission dynamics of the disease. Consequently, estimation of parameters such as the serial interval and basic reproduction number (R_0_) is being used to guide control strategies and enable disease forecasting [3-5].

In the early phase of the COVID-19 pandemic, India adopted a policy of universal health facility–based isolation of all individuals infected with SARS-CoV-2 irrespective of symptomatic status. However, in view of the increasing number of COVID-19 cases, home isolation of asymptomatic and mild cases was introduced on May 10, 2020 [6]. Due to the emerging nature of the outbreak and the evolving control measures, it is important to achieve a detailed epidemiological understanding of the COVID-19 situation at the district level to guide control measures and surge preparedness on a real-time basis.

Current mathematical modeling approaches for epidemiological understanding of COVID-19 in India are based on aggregate data reported at the national and state levels [7-13]. Very often, conclusions based on large-scale data are not appropriate for designing interventions at the local level. Therefore, we aimed to study the transmission of COVID-19 at the district level by estimating the serial interval and to determine the most suitable method for R_0_ estimation to support decision-making at the district level. We also aimed to demonstrate the feasibility of epidemic projection to guide COVID-19 response.

We studied the COVID-19 outbreak in the Jodhpur District of the state of Rajasthan in India. This mid-sized cultural and tourism hub is known as the gateway to the Thar Desert area in the northwestern part of India. Based on projection of 2011 census data to 2020 while assuming constant annual exponential growth, Jodhpur District has a population of 4.6 million, with an urban population of 1.6 million [14]. The first COVID-19 case in this district was reported on March 9, 2020, and at least one case has been reported daily since March 30, 2020.

## Methods

### Overview

We conducted a prospective observational study of the COVID-19 outbreak in Jodhpur, India. We used two data sources for the study. Firstly, serial intervals were estimated based on contact history of laboratory confirmed SARS-CoV-2 infected individuals. Secondly, the publicly available daily case count data were used together with the serial intervals to estimate R_0_ and project the size of the epidemic over the next 30 days. Individuals meeting the definition of a suspected case of COVID-19 were tested with the real time reverse transcriptase–polymerase chain reaction (rRT-PCR) at our institute in Jodhpur, India, as per national guidelines [15]. People who tested positive for SARS-CoV-2 were further assessed for their contact history with known COVID-19 cases in their household. The serial intervals were estimated based on the length of time between the onset of symptoms of the identified infectors and infectees. For asymptomatic individuals, the date of collection of the first positive sample was taken as a proxy of symptom onset.

The basic reproduction number (R_0_) is defined as the average number of susceptible individuals infected by a single primary case [16]. For R_0_ estimation, serial interval values along with daily case count data were taken from the official daily report released by the Jodhpur District administration. These data are also available on the internet [17].

### Ethical Approval

Informed consent was obtained prior to eliciting contact history for serial interval estimation. The study was approved by the Institutional Ethics Committee (Ref: AIIMS/IEC/2020-21/3047).

### Serial Interval Estimation

The mean (SD) of the serial intervals was calculated. Further, the serial interval data were fitted to weibull, log-normal, log-logistic, and generalized gamma distributions using the Flexsurv package in R software version 4.0.0 [18]. The estimates of the median serial interval were taken from the best-fitting model based on the minimum Akaike information criterion (AIC) value. The standard maximum likelihood approach was used to obtain the best model fit to the actual data.

### Estimation of R_0_

The daily COVID-19 case data in Jodhpur District were converted to incidence objects using the Incidence package in R software [19]. The EarlyR and EpiEstim packages in R were used to estimate the overall and instantaneous values of R_0_, respectively, using the parameter estimates of the serial interval [20,21]. We used two main standard methods of estimation of the instantaneous R_0_ values to visualize their response to changes in case trends and to assess their utility for understanding real-time transmission dynamics at a local level. These methods use different mathematical modeling principles and assumptions.

Instantaneous R_0_ values were first calculated using the method of estimating daily incidence based on a Poisson process determined by daily infectiousness, as proposed by Jombart et al [19] and Nouvellet et al [22]. Here, *λ_t_*, the force of infection observed on day t, is expressed by the following equation:

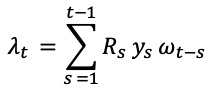



where *y_s_* is the incidence of cases on day *s* and *R_s_* is the instantaneous reproduction number on day *s*. The value of *ω_t-s_* is the probability mass distribution of the serial interval, which represents the infectiousness of incident cases on day *s* to result in secondary cases on day *t*. In the absence of an exhaustive symptomatic history of each reported case, we approximated the day the case was reported as the day of onset, a practical approach used in earlier studies [22].

Next, we used a method described by Wallinga and Teunis [23] to estimate the time-varying R_0_ based on the probability of transmission between infector-infectee pairs. We adopted the parametric method of specifying the mean (SD) of the serial interval distribution for both methods. Time windows of 7 days and 14 days were used to calculate the instantaneous R_0_.

### Forecasting of the Epidemic Size

The numbers of daily and cumulative COVID-19 cases for the next 30 days were forecasted based on the overall R_0_ value and the R_0_ value for the past 30 days as input parameters using the projections package in R [19]. The observed serial interval distribution was specified as the scale and shape parameters of the gamma distribution. Daily COVID-19 cases were predicted based on a Poisson process determined by daily infectiousness [22]. The specified serial interval distribution was taken as a prior while using the Bayesian methodology for Markov chain Monte Carlo sampling using the Metropolis algorithm. The 95% CIs of the projected daily and cumulative incidences were calculated using the bootstrap resampling method with 1000 samples.

Further, we considered two scenarios: one with a reasonable reduction of 25% SARS-CoV-2 transmission and one with an aggressive reduction of 50% transmission. A reasonable reduction would be related to compliance with strengthening of existing measures, such as contact tracing, testing, and prompt isolation of infected individuals along with physical distancing measures. Aggressive transmission reduction measures included universal mask-wearing and measures to reduce outdoor transmission through prevention of gatherings: closures of places of worship, marketplaces, restaurants, schools, and gymnasiums, along with introduction of nighttime curfews [24].

## Results

### Serial Interval

**Figure 1:**
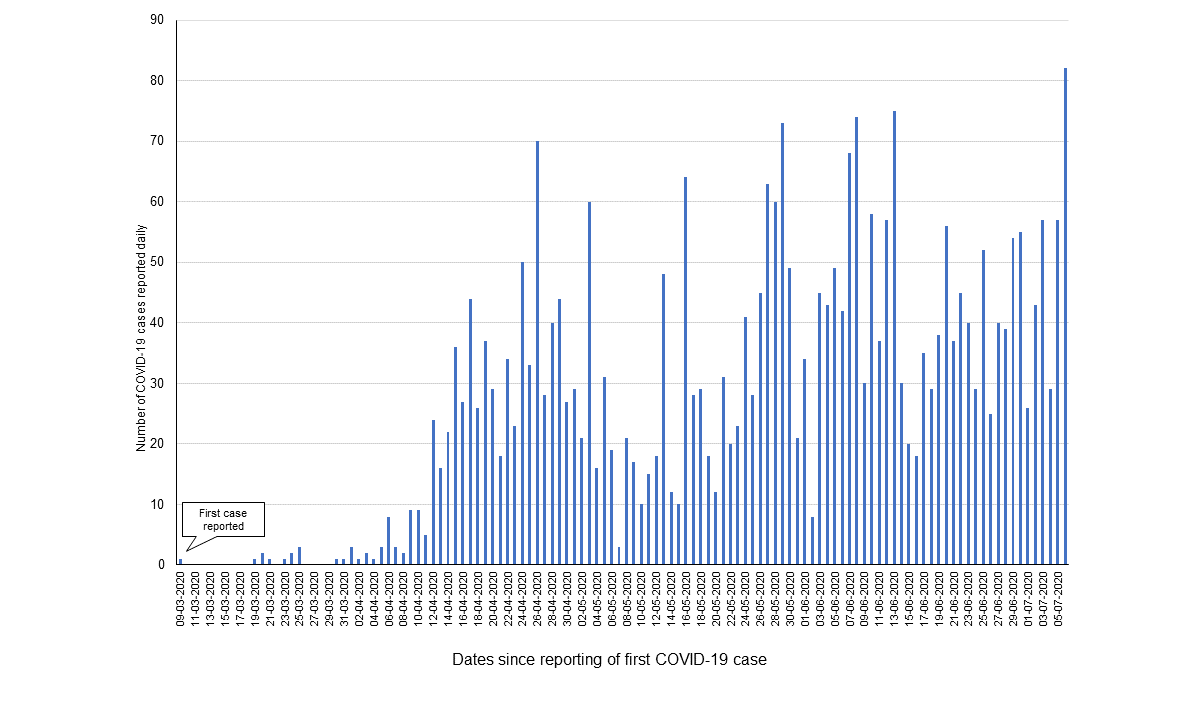
Numbers of COVID-19 cases reported daily in Jodhpur, India, from March 9 to July 6, 2020.

From the reporting of the first case of COVID-19 in Jodhpur District on March 9, 2020, to July 6, 2020, 3178 cases were reported in the district in a span of 120 days (see Figure 1). Serial interval data for 103 infector-infectee pairs were obtained through contact tracing of known infected cases (Multimedia Appendix 1).

The mean serial interval was 6.23 days (SD 3.49). The generalized gamma distribution was found to best fit the serial interval and showed the minimum AIC value (see Table 1).

**Table 1:** Fits of weibull, log-normal, log-logistic, and generalized gamma distributions with the serial interval data for SARS-CoV-2 infection in Jodhpur District, India, and the estimated median and 95 percentile values (N=103 pairs).

Type of distribution used in the model	–2 log-likelihood	Number of model parameters (k)	AIC^a^ (–2 log-likelihood + 2k)	Serial interval (days)	
				Median (95% CI)	95th percentile (95% CI)
Weibull	520.30	2	524.30	5.83 (5.18-6.54)	12.51 (11.28-13.95)
Log-normal	488.55	2	492.55	5.40 (5.06-6.07)	11.96 (10.43-13.82)
Log-logistic	492.46	2	496.46	5.44 (4.96-5.98)	12.15 (10.44-14.39)
Gamma	500.15	2	504.15	5.77 (5.23-6.33)	11.79 (10.52 -13.22)
Generalized gamma	482.97	3	488.97	5.23 (4.72-5.79)	13.20 (10.90-18.18)

^a^AIC: Akaike information criterion.

**Figure 2:**
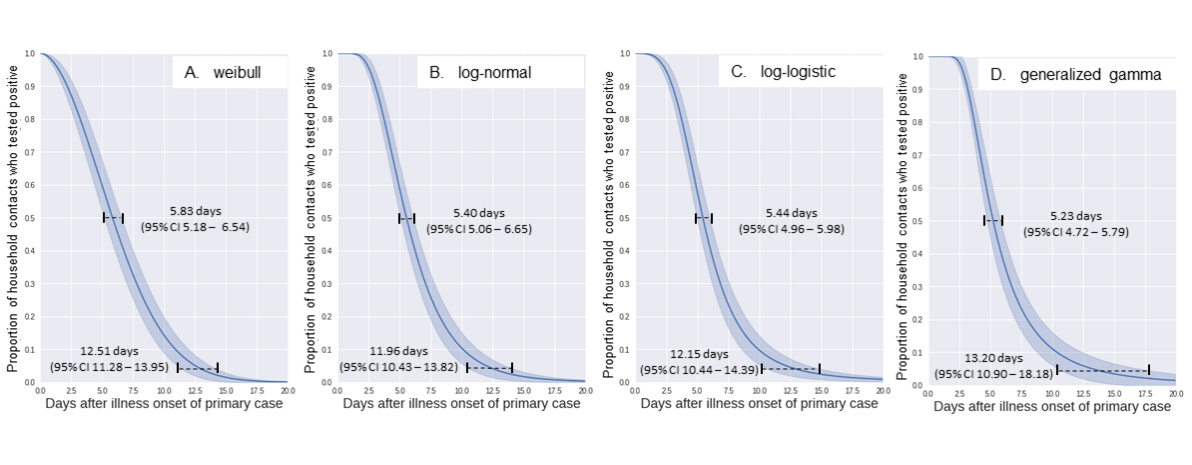
Estimates of the median and 95th percentile of the serial interval data fitted to (A) weibull, (B) log-normal, (C) log-logistic, and (D) generalized gamma distributions (N=103 pairs).

The median and 95th percentile values of the serial interval were 5.23 days (95% CI 4.72-5.79) and 13.20 days (95% CI 10.90-18.18), respectively, estimated from the fitted generalized gamma distribution (see Figure 2).

### Estimation of R_0_

The overall R_0_ value in the 30 days after the first case was reported was 1.62 (95% CI 1.07-2.17), which subsequently decreased to 1.15 (95% CI 1.09-1.21). The overall R_0_ value for the entire outbreak duration was 1.07 (95% CI 1.04-1.11), whereas it was 1.20 (95% CI 1.14-1.27) in the last 30 days.

**Figure 3:**
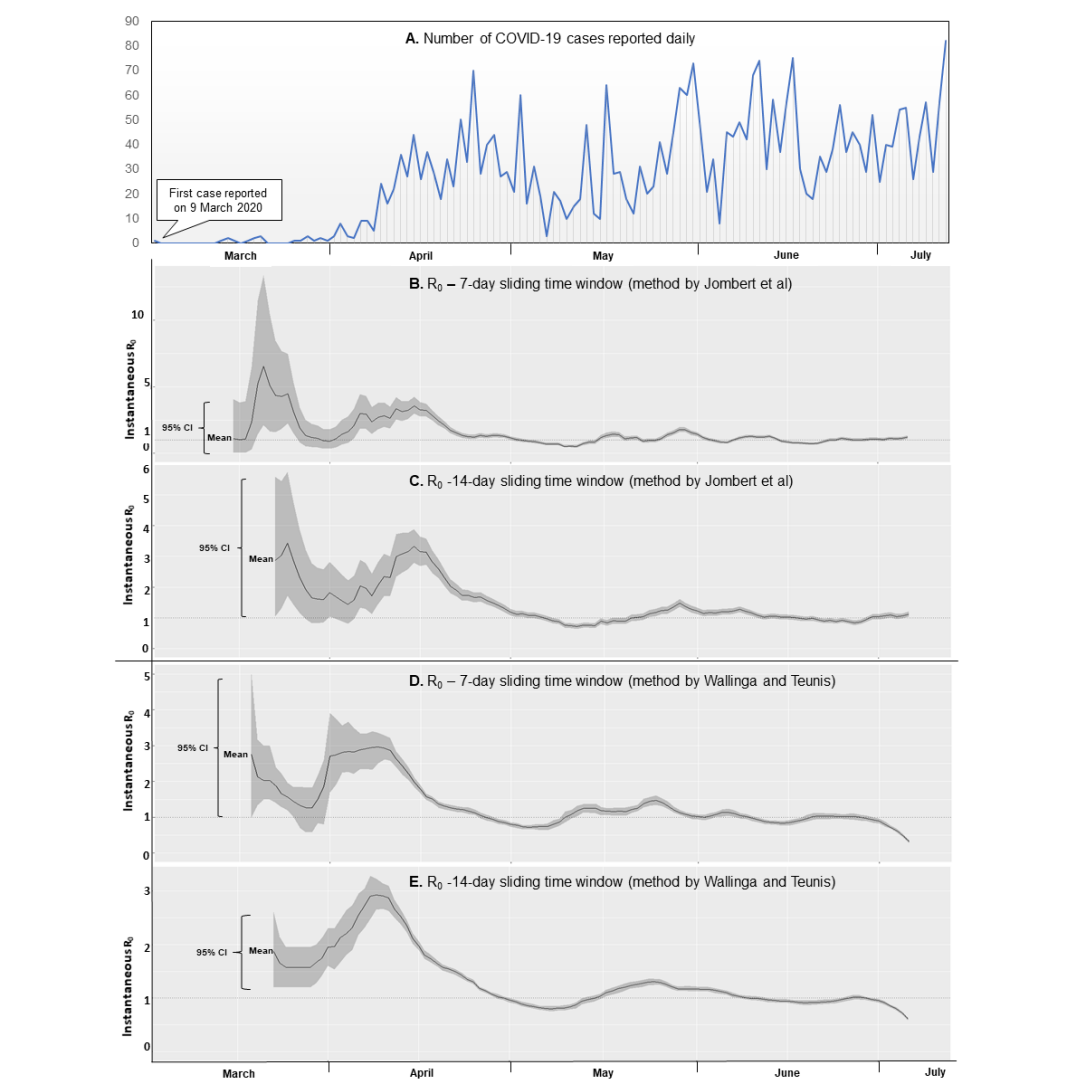
Daily COVID-19 cases in Jodhpur District, India, from March 9 to July 6, 2020 (A), instantaneous R_0_ values estimated using the method by Jombart et al (B-C), and instantaneous R_0_ values estimated using the method by Wallinga and Teunis using time windows of 7 and 14 days, respectively (D-E).

The time-dependent instantaneous R_0_ values calculated using the method by Jombart et al [19] and Nouvellet et al [22] yielded maximum values of 6.53 (95% CI 2.12-13.38) and 3.43 (95% CI 1.71-5.74) using sliding time windows of 7 days and 14 days, respectively (see Table 2 and Figure 3). Similarly, using the method described by Wallinga and Teunis [23], the maximum values of the instantaneous R_0_ were 2.96 (95% CI 2.52-3.36) and 2.92 (95% CI 2.65-3.22) for the 7- and 14-day time windows, respectively (see Table 2 and Figure 3). The peak R_0_ values corresponded with the daily rising trend in COVID-19 cases that was reported (see Figure 3). 

The latest instantaneous R_0_ values estimated on July 6, 2020, using the method by Jombart et al [19] and Nouvellet et al [22], were 1.21 (95% CI 1.09-1.34) and 1.12 (95% CI 1.03-1.21) for 7- and 14-day sliding time windows, respectively (see Table 2 and Figure 3). Similarly, the latest instantaneous R_0_ values estimated on July 6, 2020, using the method by Wallinga and Teunis [23] were 0.32 (95% CI 0.27-0.36) and 0.61 (95% CI 0.58-0.63), for the 7- and 14-day sliding time windows, respectively (see Table 2 and Figure 3).

**Table 2:** Summary of the time-dependent R_0_ values estimated by the different methods.

Method used and sliding time windows		Minimum value (95% CI)	Maximum value (95% CI)	Latest value as of July 6, 2020 (95% CI)
**Jombart et al and Nouvellet et al**				
	7-day	0.52 (0.42-0.62)	6.53 (2.12-13.38)	1.21 (1.09-1.34)
	14 day	0.72 (90.64-0.80)	3.43 (1.71-5.74)	1.12 (1.03-1.21)
**Wallinga and Teunis**				
	7-day	0.32 (0.27-0.36)	2.96 (2.52-3.36)	0.32 (0.27-0.36)
	14-day	0.61 (0.58-0.63)	2.92 (2.65-3.22)	0.61 (0.58-0.63)

### Projection of Epidemic Size

**Figure 4:**
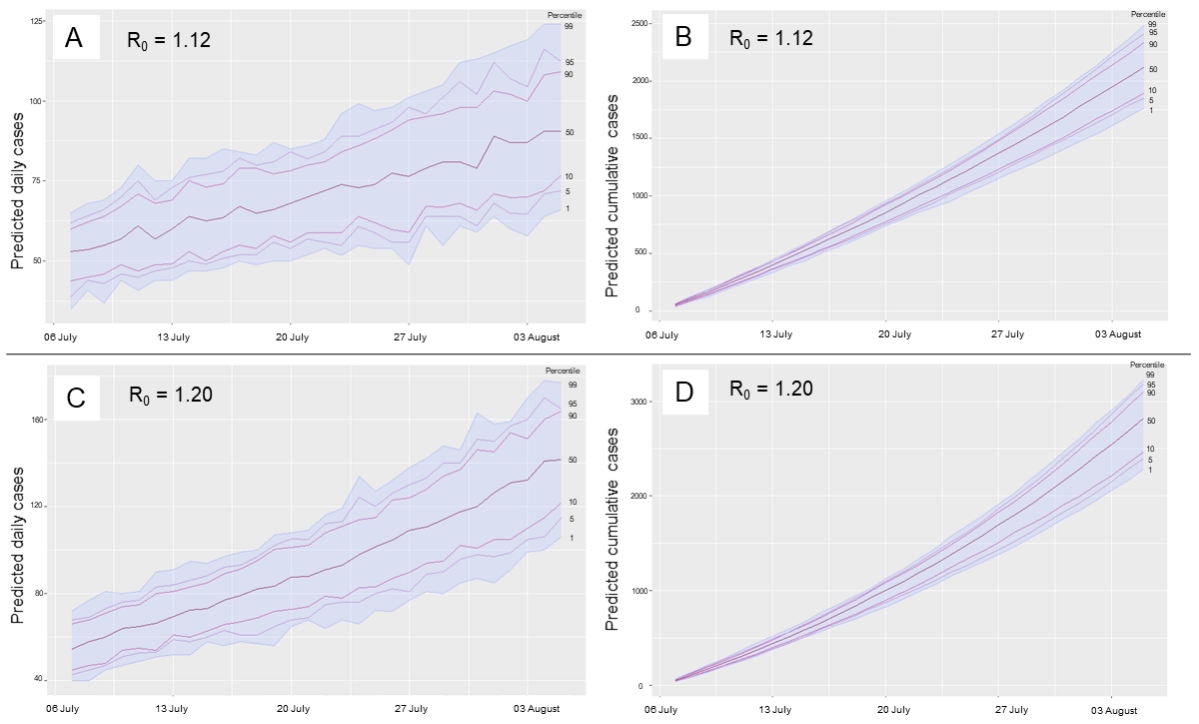
Projections of daily and cumulative COVID-19 caseloads over the next 30 days using the instantaneous R_0_ value of 1.12 on July 6, 2020 (A-B) and the overall R_0_ value of 1.20 for the most recent 30 days (C-D).

The number of daily cases projected for the next month based on an overall R_0_ value of 1.20 (corresponding to the most recent 30 days of transmission) ranged from 55 (95% CI 38-71) on July 7, 2020 (day 1), to 143 (95% CI 110-175) on August 5, 2020, (ie, on day 30; see Figure 4). Similarly, the number of daily cases projected for the next month while taking the most recent 14-day rolling instantaneous R_0_ value of 1.12 ranged from 52 (95% CI 38-66) on day 1 to 91 (95% CI 66-116) on day 30 (see Figure 4). The cumulative projections of the number of COVID-19 cases over the next 30 days using the R_0_ values of 1.20 and 1.12 were 2817 (95% CI 2374-3259) and 2131 (95% CI 1799-1462), respectively.

The scenarios of 25% and 50% transmission reduction of the most recent time-dependent R_0_ estimate (ie, reduction of R_0_ from 1.12 to 0.84 and 0.56, respectively) resulted in monthly projections of 880 cases (95% CI 699-1061) and 341 cases (95% CI 265-418); these projections correspond to 58.7% and 84.0% reductions in the epidemic size in Jodhpur, respectively.

## Discussion

### Implications of the Serial Interval and Estimated R_0_ Values

Our observation of the mean serial interval fell within a range of 4 to 8 days, as estimated by a meta-analysis of 7 studies conducted during the early phase of the COVID-19 pandemic [25]. Another meta-analysis including studies only from China estimated a range of serial intervals from 4.10 to 7.5 days [26]. Our experience suggests that the median and 95% CI estimates of the serial interval should be reported alongside the mean and SD, as the latter approach is more susceptible to influence by extreme values. It has also been suggested that longer serial interval intervals may be due to preventive interventions introduced during the course of the epidemic, which tend to reduce transmission [27,28]. Therefore, it is preferable to estimate the recent serial interval at a local level to better understand the transmission of SARS-CoV-2.

The distribution of R_0_ values was consistent with observations from other countries, indicating a similar transmission pattern [4,28]. The peak of the R_0_ value was reached in the first week of April 2020. The subsequent reduction toward the end of April can be attributed to aggressive testing, contact tracing, and isolation measures implemented in the urban area of Jodhpur during that month. Our R_0_ estimate for the first month (1.61) was slightly higher than the national estimate of 1.47 and lower than the estimate from the state of Tamil Nadu (1.88) in India during the same period of March to April 2020 [9,13]. District level R_0_ estimates are more likely to show pronounced fluctuations than state or national estimates, as the latter are aggregated across a wide range of epidemiological settings. Because district-level R_0_ estimates were not available from India, we compared our findings with those from the cities of Qom and Shahroud in Iran [29,30]. Similar to these cities, Jodhpur showed a trend of high values of R_0_ in the first 14-30 days, with a subsequent decrease toward 1 [29,30]. The initially high R_0_ values can be attributed to the suddenness of the outbreak if the surveillance system is robust. The high values may also be due to a sudden start of case reporting following an initial period of underreporting, leading to an artefactual peak in R_0_ [30].

Earlier detection of infection followed by isolation is known to reduce the R_0_ value by limiting both the duration of effective contact and the number of susceptible people an infected individual can come in contact with [16]. Our findings further support that parameters such as the serial interval, incubation period, and R_0_ value are likely to vary throughout the course of the epidemic and will depend on local factors influencing transmission, such as demographics, environmental conditions, modeling methodology, and stringency of control measures [16,30].

### Epidemic Projections

The projected estimate of daily cases and the final outbreak size were found to depend on the value of R_0_ entered in the model [31-33]. The method used to estimate the R_0_ value and the time window over which R_0_ was calculated both influenced the final projection by a wide margin. The 14-day time window yielded less variable instantaneous R_0_ estimates compared to the 7-day time window. We found that the method by Wallinga and Teunis was more sensitive to recent fluctuations in daily case count than the method by Jombart et al in the same time window. Further, per the renewal equation stated earlier, the values of R_0_ are most influenced by the trend in daily cases reported within the range of the serial interval (ie, within 5 to 6 days). This model also assumes homogenous mixing, which becomes less applicable with larger populations in which cases emerge from widely separated clusters. Also, the impact of the method of R_0_ estimation and the time window was more pronounced when there was a fluctuating trend in cases or when the R_0_ value was close to 1. In research settings, R_0_ values should be tested through sensitivity analyses by considering variations in time windows and durations and using different methods so that reliable projections can be provided for larger populations [31]. For routine use within program settings at the district level, the method by Jombart et al may be preferable for monitoring the effectiveness of control methods and providing prior R_0_ values for projections compared to the method by Wallinga and Teunis and the overall R_0_ estimation using the EarlyR package in R.

Our study was based on contact history of infected individuals instead of on daily follow-up of contacts of infected individuals for disease onset. Therefore, we minimized underreporting of longer serial intervals, which may be due to right-truncation in the follow-up method for assessing the serial interval [30]. Further, the use of the time-varying method for daily R_0_ estimation and the maximum likelihood method for overall R_0_ estimation has the benefit of lower bias compared to the exponential growth and sequential Bayesian methods [34]. It also enables assessment of the effectiveness of control measures on a real-time basis, in contrast to other methods that only provide an aggregate R_0_ value [30].

### Limitations

One limitation of our study is that population level estimates relying on daily official reports can underestimate the value of R_0_ compared to those of closed populations because many infected individuals are likely to be missed, especially if the testing capacity is limited or the proportion of asymptomatic people is high [31]. Further, modeling assumptions such as assuming a finite probability of interaction of infector-infectee pairs reported within a serial interval range may not be applicable for large population cohorts [23]. To overcome these limitations, use of both spatial and temporally structured data has been proposed [35]. The use of contact tracing applications that provide anonymized geolocated data and serial interval estimates could provide more timely and robust epidemiological understanding of emerging diseases such as COVID-19 [36,37].

### Conclusions

Public health measures such as testing, contact tracing, and home isolation were found to reduce the instantaneous R_0_ value and could thereby reduce the final outbreak size. Instantaneous R_0_ estimated using the method proposed by Jombart et al is recommended for guiding COVID-19 response strategy at district level in preference to the method proposed by Wallinga and Teunis and to aggregate R_0_ calculation. The final epidemic size was found to be influenced by the R_0_ value, which in turn depended on the stringency of control measures. Even a marginal reduction in R_0_ as a result of strengthening control measures was found to considerably reduce the projected COVID-19 burden at the district level. Projections based on publicly released daily COVID-19 case data are feasible and could be useful in guiding a data-driven COVID-19 response strategy at a local level. This could be used for both surge capacity planning of the number of hospital beds and ventilators required and for public health responses such as the number of staff required for contact tracing and for provisioning of institutional quarantine or isolation facilities. Therefore, considering the increasing caseload and dynamic situation of COVID-19, a decentralized evidence-driven approach is currently needed.

## Appendix

Multimedia Appendix 1 Data used for the serial interval estimation of COVID-19 in Jodhpur, India (103 pairs).

## Abbreviations

AIC: Akaike information criterion
R_0_: basic reproduction number
rRT-PCR: real-time reverse transcription–polymerase chain reaction
